# The Negative Impacts of Acromegaly on Bone Microstructure Not Fully Reversible

**DOI:** 10.3389/fendo.2021.738895

**Published:** 2021-09-15

**Authors:** Lian Duan, Shengmin Yang, Lin Jie Wang, Yuelun Zhang, Ran Li, Hongbo Yang, Yuxing Zhao, Hanze Du, Xiao Zhai, Fengying Gong, Hui Pan, Huijuan Zhu, Weibo Xia

**Affiliations:** ^1^Key Laboratory of Endocrinology of National Health Commission, Department of Endocrinology, State Key Laboratory of Complex Severe and Rare Diseases, Peking Union Medical College Hospital, Chinese Academy of Medical Science and Peking Union Medical College, Beijing, China; ^2^Medical Research Center, Peking Union Medical College Hospital, Chinese Academy of Medical Sciences and Peking Union Medical College, Beijing, China

**Keywords:** acromegaly, bone microstructure, high-resolution peripheral quantitative computed tomography, disease activity, bone turnover, GH, IGF-1

## Abstract

**Purpose:**

This study aimed to evaluate the bone turnover markers and bone microarchitecture parameters derived from high-resolution peripheral quantitative computed tomography (HR-pQCT) in active and controlled acromegaly patients.

**Methods:**

This cross-sectional study involved 55 acromegaly patients from a tertiary hospital (23 males and 32 females, aged 45.0 ± 11.6 years). Firstly, growth hormone (GH), insulin-like growth factor-1 (IGF-1), and markers for bone turnover were assessed. Next, we derived peripheral bone microstructure parameters and volumetric bone mineral density (vBMD) through HR-pQCT. These parameters were compared between acromegaly patients and 110 healthy controls, as well as between 27 active and 28 controlled acromegaly patients. Moreover, the relationship between GH/IGF-1 and bone microstructure parameters was analyzed through multiple linear regression.

**Results:**

As compared with healthy controls, acromegaly patients exhibited elevated cortical vBMD, reduced trabecular vBMD, and increased trabecular inhomogeneity in the distal radius and tibia. While controlled acromegaly patients had slower bone turnover, they did not necessarily have better bone microstructure relative to active patients in intergroup comparison. Nevertheless, multiple regression indicated that higher IGF-1 was associated with lower tibial stiffness and failure load. Additionally, males with higher IGF-1 typically had larger trabecular separation, lower trabecular number, and larger cortical pores in the radius. Moreover, patients with elevated GH typically had more porous cortical bone in the radius and fewer trabeculae in the tibia. However, the compromised bone strength in active patients was partially compensated by increased bone thickness. Furthermore, no significant linkage was observed between elevated GH/IGF-1 and the most important HR-pQCT parameters such as trabecular volumetric bone density.

**Conclusion:**

Acromegaly adversely affected bone quality, even in controlled patients. As the deterioration in bone microstructure due to prolonged GH/IGF-1 exposure was not fully reversible, clinicians should be aware of the bone fragility of acromegaly patients even after they had achieved biochemical remission.

## Introduction

Acromegaly is characterized by elevated growth hormone (GH) and insulin-like growth factor-1 (IGF-1), most commonly caused by pituitary adenoma. Persistently elevated GH/IGF-1 may result in multiple complications, such as osteopathy, tissue hypertrophy, and metabolic disorders. Acromegaly patients are susceptible to vertebral fractures even when their bone mineral density (BMD) appears normal. Moreover, such fractures may continue to progress even after the disease had been adequately controlled (1–3).

Dual-energy X-ray absorptiometry (DXA), the standard way for measuring areal BMD (aBMD), is largely constrained by its bi-dimensional nature. Specifically, degenerative joint disease, featured by the formation of osteophytes and the hypertrophy of facet joints, accounts for overestimated BMD measurements in lumbar spines. Moreover, DXA can hardly differentiate trabecular bone from surrounding cortical bone, thereby considerably affected by the variable distributions of those two compartments at different skeletal sites (4). Actually, the quality and weight-bearing capacity of bones mainly depend on the trabecular microstructure but not on cortical bone thickness. In acromegaly patients, although the trabecular microarchitecture remarkably deteriorated, the cortical bone thickness increased, rendering deceptively normal aBMD measurements (5).

The abovementioned pitfalls of DXA can be avoided by performing high-resolution peripheral quantitative computed tomography (HR-pQCT) in the distal tibia and radius. HR-pQCT is characterized by higher spatial resolution and superior bone structural delineation and generates near-isotropic volumetric datasets (6). Apart from bone microarchitecture parameters, bone strength and stiffness can be estimated from these images. Particularly, mechanical performance of bones can be calculated by the finite element analysis (FEA), which predicts bone response to stimulating loads based on structural data derived from HR-pQCT images.

Acromegaly was related to chaotic bone turnover and an elevated risk for fragility fractures, especially when untreated hypogonadism coexisted (7). Additionally, in an Italian cohort (n = 40), acromegaly patients with radiological vertebral fractures suffered from decreased trabecular bone volume ratio, elevated trabecular separation (Tb.Sp), and increased cortical porosity (Ct.Po) (8). Up until now, the bone microstructure of acromegaly subjects had rarely been investigated through HR-pQCT in Asia. The present study was designed to quantify bone microarchitecture of Chinese acromegaly patients through HR-pQCT, evaluate bone remodeling markers, and explore the effects of excessive GH/IGF-1 exposure on bone quality.

## Methodology

### Subjects

Fifty-five acromegaly patients who attended our outpatient clinic at the Peking Union Medical College Hospital (PUMCH) were consecutively enrolled in this study from May 2019 to November 2019. All patients were diagnosed by the following criteria: 1) IGF-1 beyond the age-specific reference range and GH ≥1 ng/ml during the oral glucose tolerance test (OGTT) before receiving any treatments, 2) the presence of a pituitary tumor in MRI/CT scan, and 3) clinical manifestations (9). In this study, if a patient’s IGF-1 fell below the sex- and age-specific upper limit of normal (ULN) or GH nadir in OGTT/random GH was below 1 ng/ml, then the diagnosis of “controlled” acromegaly was made. Otherwise, the disease status for this patient was marked as active. With regard to the gonadal status of patients, males were considered eugonad if total testosterone was at least 3 ng/ml (10). Females were considered eugonad if they had regular menstrual periods or they were on sex hormone replacement therapy. Regular menstruation was defined by low inter-cycle variation (≤9 days). Additionally, the frequency of menstruation was considered normal if the menstruation interval lasted between 24 and 38 days (11). The diagnosis of menopause was retrospectively made based on the cessation of menses for at least 12 months in a female with uterus. Women who had undergone a hysterectomy but not bilateral oophorectomy were considered to have ovarian failure and marked as menopausal if elevated follicle-stimulating hormone (FSH) concentrations and low estradiol levels (<20 pg/ml) were consistently observed during clinical visits (12). In addition, normal subjects matched for sex, age, and body mass index (BMI) were selected in a ratio of 1:2 from healthy non-athletic people recruited through posters in the physical examination center from June 2015 to February 2017.

The inclusion criteria for acromegaly patients were as follows: 1) diagnosed of acromegaly according to the abovementioned criteria; 2) 18 to 70 years old; and 3) no previous fractures on at least one side of the radius and tibia (to facilitate unbiased HR-pQCT assessment). The exclusion criteria were as follows: 1) persisting metabolic bone disease other than postmenopausal bone loss, including Paget’s disease, osteomalacia, hyperparathyroidism or hypoparathyroidism; 2) untreated anterior hypopituitarism; 3) use of medications known to affect bone metabolism, such as anti-osteoporotic drugs and glucocorticoids, with the exception of sex hormone replacement, levothyroxine (as long as free triiodothyronine (FT3), free thyroxine (FT4), and thyroid-stimulating hormone (TSH) were kept within normal ranges), vitamin D, and calcium; 4) severe liver damage (serum alanine aminotransferase ≥two-fold of ULN); 5) severe kidney disease (serum creatinine level ≥150 μmol/L); 6) diagnosis of cancer or multiple endocrine neoplasia (MEN); 7) chronic drug abuse and/or alcohol abuse; and 8) chronic gastrointestinal disorders. Our study protocol was approved by the Institutional Review Committee at PUMCH. Each participant had provided written informed consent before enrollment.

### Anthropometric Data Collection

The standing height of each subject was measured to the nearest 0.1 cm by a digital stadiometer (Hampton, Seritex, East Rutherford, NJ, USA). The body weight was recorded to the nearest 0.1 kg by the RGZ-120 digital electronic weight scale (Xiheng, China). The BMI was calculated as weight (kg)/height (m^2^).

### Biochemical Assays

Fasting venous blood was sampled on the date of bone evaluation between 8:00 and 9:00 AM. Sex hormones, thyroid hormones, serum phosphate, serum calcium (Ca), HbA1c, and alkaline phosphatase (ALP) were routinely measured at the Department of Clinical Laboratory at PUMCH with standardized methods. Total procollagen I N-terminal peptide (PINP), 25-hydroxyvitamin D [25(OH)D], β-C-terminal telopeptide of type 1 collagen (β-CTX, quantified *via* β-CrossLaps assay), and parathyroid hormone (PTH) were measured through Elecsys reagent kits (Roche Diagnostics, Basel, Switzerland). GH and IGF-1 were determined by the full automatic, solid-phase, two-site, chemiluminescent enzyme immunometric assay (Immulite 2000, Siemens Healthcare Diagnostics, GH calibrated against the recommended IS 98/574). Moreover, standard 2-h 75-g OGTT was performed in each patient after overnight fasting.

### Dual-Energy X-Ray Absorptiometry and Thoracolumbar Spine X-Ray

This study applied DXA (Lunar Prodigy Advance, GE, USA) in measuring the aBMD of total hip, femoral neck, and lumbar vertebra (L1–L4) at the posteroanterior position. The aBMD was calculated through dividing the bone mineral content (BMC) by the projected area of the scanned image [BMD = BMC/area (g/cm^2^)]. In men over 50 years old and postmenopausal women, osteoporosis was diagnosed when the T-score [standard deviation difference (SDS) based on peak bone mass in young subjects with same sex and race) was ≤−2.5 SD in the spine or hip, whereas osteopenia was diagnosed when the T-score was between −1 and −2.5 SD. However, as for men below 50 years old and women before menopause, aBMD was considered “below the expected range for age” when the Z-score (SDS from sex-, race-, and age-matched controls] was ≤−2.0 SD (13). Lateral thoracolumbar X-ray was performed to detect vertebral fractures in each patient, using the semiquantitative approach introduced by Genant et al. (14). All images were reviewed by two experienced radiologists in a blinded manner, and disputes were resolved through discussion after independent grading had been finished. In this study, radiographic vertebral fracture was diagnosed if ≥20% reduction in vertebral height relative to nearby vertebrae was observed (grade 1: mild fracture, reduction in vertebral height between 20% and 25%; grade 2: moderate fracture, reduction in vertebral height between 26% and 40%; and grade 3: severe fracture, reduction in vertebral height over 40%) (14).

### High-Resolution Peripheral Quantitative Computed Tomography and Finite Element Analysis

HR-pQCT scanning was conducted on the non-dominant distal radius and ipsilateral tibia, unless there was a previous fracture at the target site (XtremeCT II scanner, ScancoMedical, Brüttisellen, Switzerland) (15). Each subject was scanned according to the standard protocol *in vivo* (1,000 µA, 60 kVp, and integration time of 100 ms). Firstly, a scout view was taken to locate the desired measurement region and to put the reference lines at the end plates of the distal radius or tibia. Next, 110 parallel slices were acquired simultaneously at a resolution of 82 µm over an area of 10.2 mm with the first slice placed at 9.0 mm (radius) or 22.0 mm (tibia) away from the respective proximal reference line (16). Then, the scans were examined for motion artifacts and graded on a scale of 1 (no motion) to 5 (worst quality), and images graded ≥3 were excluded from the analysis (17). Afterwards, bone microarchitecture parameters, including total area (Tt.Ar), cortical area (Ct.Ar), trabecular area (Tb.Ar), total volumetric BMD (vBMD) (Tt.vBMD), cortical vBMD (Ct.vBMD), trabecular vBMD (Tb.vBMD), trabecular number (Tb.N), Tb.Sp, trabecular thickness (Tb.Th), trabecular network inhomogeneity (Tb.1/N.SD), and trabecular bone volume fraction [bone volume/tissue volume (Tb.BV/TV)] were acquired through normalized morphological analyses following the workflow proposed by Boutroy et al. (16). In addition, we used an automated segmentation algorithm [Image Processing Language (version 5.08b, Scanco Medical)] to quantify Ct.Po, cortical thickness (Ct.Th), and cortical perimeter (Ct.Pm). Moreover, in order to calculate the bone stiffness (N/mm), we transformed the HR-pQCT images into finite element models by FAIM (version 6.0, Numerics88, Calgary, Canada) (18). Furthermore, the Pistoia criterion was applied in the estimation of the bone failure load (N) (19). In our laboratory, the tolerance for error in density measurements was ±8 mg HA/cm^3^, which corresponds to 1% error for a cylindrical 800 mg HA/cm^3^ insert. In addition, the maximum allowable root mean squared coefficient of variation (RMSCV) for bone morphologic parameters was 2%.

### Statistical Analysis

The frequencies and percentages were shown for categorical variables, whereas the numerical variables were presented as the mean ± SD or median (interquartile range). The distribution of continuous variables was examined by the Shapiro–Wilk test and q-q plot. The equality of variances between the two groups in comparison is evaluated by Levene’s test. For normally distributed data with roughly equal intergroup variances, unpaired *t*-test was performed for intergroup comparisons. The data were ln-transformed if the assumption for normality or equality of variances was violated. If the transformed data had roughly equal intergroup variances, unpaired *t*-test was applied. The resultant p-value was recorded and reported together with the mean and SD for the original data. Otherwise, the Mann–Whitney U test was employed to assess the difference between these two groups. In addition, differences in proportions were analyzed using Yates’s continuity corrected chi-square test or Fisher’s exact test (when at least one cell in the contingency table had cell count < 5). Moreover, we performed multiple linear regression to quantify the relationship between IGF-1 level and bone microstructure parameters after assessing Pearson’s correlation coefficient between potential independent variables. We controlled for parameters that were significantly related to both IGF-1 level and bone microarchitecture parameters in regression and inspected the residual plot for each regression model. The abovementioned computations were run using SPSS (IBM SPSS Statistics for Macintosh, Version 26.0. Armonk, NY: IBM Corp.), and a p-value <0.05 was considered statistically significant.

## Results

### Demographics and Clinical Features

Fifty-five acromegaly patients (23 males and 32 females, median age 45.0 ± 11.6 years) were included in this study. As shown in **Table 1**, one patient was at the stage of initial diagnosis and treatment-naïve, 14 patients were treated by surgery alone, seven patients were on somatostatin analogs (SSAs) alone, three patients underwent surgery and radiotherapy, 14 patients were treated by surgery and SSAs, three patients received radiotherapy and SSAs, and 13 patients received all three therapies (surgery + radiotherapy + SSAs). Twenty-seven patients manifested active acromegaly, while the disease activity was controlled in 28 patients. With regard to the gonadal status of acromegaly women, five women in the active group (average age at menopause: 52.0, average accumulative years since menopause: 7.5) and eight women in the stable group (average age at menopause: 51.4, average accumulative years since menopause: 4.3) were menopausal. One women in the active group and four in the control group had central hypogonadism, but all of them were on combined estrogen–progestin therapy (2 mg of estradiol valerate for 11 days, followed by 2 mg of estradiol valerate plus 1 mg of cyproterone acetate for 10 days), which induced periodical vaginal bleeding. These women on standardized sex hormone replacement therapy were considered to be eugonad in our study. In terms of aBMD, 42 patients were examined by DXA, among whom one was diagnosed with osteoporosis, four were diagnosed with osteopenia, and three had aBMD “below the expected range for age” according to the abovementioned diagnostic criteria (13). Thoracolumbar spine X-ray was performed in 33 patients, among whom five had vertebral fractures.

**Table 1:** Clinical characteristics of 55 acromegaly patients.

		Acromegaly patients (n = 55)
Age (years)		45.0 ± 11.6
Gender (male/female)		23/32
BMI (kg/m^2^)		25.4 ± 3.9
Duration of disease (years)		12.4 ± 8.2
Tumor size (micro-/macroadenoma)		4/51
Therapy		
Treatment naive		1
Surgery only		14
SSAs only		7
Surgery combined with radiotherapy		3
Surgery combined with SSAs		14
Radiotherapy combined with SSAs		3
Surgery combined with radiotherapy and SSAs		13
Diabetes mellitus (%)		38.2 (21/55)
DXA measurements (%)	T-score ≤ −2.5SD	7.1 (1/14)
	−2.5SD < T-score < −1SD	28.6 (4/14)
	Z-score ≤ −2SD	10.7 (3/28)
Vertebral fractures detected by thoracolumbar X-ray (%)		9.1 (3/33)

BMI, body mass index; SSA, somatostatin analog; DXA, dual-energy X-ray absorptiometry.

### Changes in the High-Resolution Peripheral Quantitative Computed Tomography Bone Microstructure Parameters in Acromegaly Patients Relative to Healthy Controls

In this study, 55 acromegaly patients and 110 healthy volunteers were included. Patients and controls were comparable with regard to age (45.0 ± 11.6 *vs.* 44.9 ± 13.1 years, p *=* 0.978) and BMI (25.4 ± 3.9 *vs.* 25.6 ± 4.0 kg/m^2^, p *=* 0.811).

As for the distal radius (**Table 2**), acromegaly patients had significantly increased Tt.vBMD (390.16 ± 89.29 *vs.* 326.06 ± 68.93 mg HA/cm^3^, p < 0.001), Ct.vBMD (953.69 ± 59.16 *vs.* 915.59 ± 65.29 mg HA/cm^3^, p < 0.001), Ct.Ar (91.91 ± 21.88 *vs.* 68.22 ± 16.12 mm^2^, p < 0.001), and Ct.Th (1.563 ± 0.347 *vs.* 1.115 ± 0.212 mm, p < 0.001). Moreover, these patients had significantly decreased Tb.Ar (193.89 ± 68.22 *vs.* 228.34 ± 64.43 mm^2^, p *=* 0.002), Tb.vBMD (103.94 ± 45.08 *vs.* 145.18 ± 46.75 mg HA/cm^3^, p < 0.001), Tb.BV/TV (0.164 ± 0.057 *vs.* 0.216 ± 0.067, p < 0.001), and Tb.N [1.039 (0.511) *vs.* 1.328 (0.337)/mm, p < 0.001], together with increased Tb.Sp [0.936 (0.514) *vs.* 0.713 (0.209) mm, p < 0.001] and Tb.1/N.SD [0.429 (0.403) *vs.* 0.274 (0.099) mm, p < 0.001].

**Table 2:** HR-pQCT parameters of the distal radius and tibia in acromegaly patients and healthy volunteers.

HR-pQCT parameters	Acromegaly patients(n = 55)	Healthy volunteers(n =110)	p-Value
Radial Tt.Ar (mm^2^)	281.96 ± 76.52	292.7 ± 72.05	0.381
Radial Tb.Ar (mm^2^)	193.89 ± 68.22	228.34 ± 64.43	**0.002**
Radial Ct.Ar (mm^2^)	91.91 ± 21.88	68.22 ± 16.12	**<0.001^#^**
Radial Tt.vBMD (mg HA/cm^3^)	390.16 ± 89.29	326.06 ± 68.93	**<0.001^#^**
Radial Tb.vBMD (mg HA/cm^3^)	103.94 ± 45.08	145.18 ± 46.75	**<0.001**
Radial Ct.vBMD (mg HA/cm^3^)	953.69 ± 59.16	915.59 ± 65.29	**<0.001**
Radial Tb.BV/TV	0.164 ± 0.057	0.216 ± 0.067	**<0.001**
Radial Tb.N (1/mm)	1.039 (0.511)	1.328 (0.337)	**<0.001^##^**
Radial Tb.Th (mm)	0.237 ± 0.028	0.232 ± 0.019	0.113
Radial Tb.Sp (mm)	0.936 (0.514)	0.713 (0.209)	**<0.001^##^**
Radial Tb.1/N.SD (mm)	0.429 (0.403)	0.274 (0.099)	**<0.001^##^**
Radial Ct.Pm (mm)	72.92 ± 10.93	71.41 ± 9.218	0.357
Radial Ct.Po (%)	0.006 ± 0.005	0.005 ± 0.004	0.36
Radial Ct.Th (mm)	1.563 ± 0.347	1.115 ± 0.212	**<0.001^#^**
Tibial Tt.Ar (mm^2^)	719.77 ± 131.44	713.06 ± 139.0	0.768
Tibial Tb.Ar (mm^2^)	579.69 ± 120.64	587.01 ± 133.63	0.734
Tibial Ct.Ar (mm^2^)	145.57 ± 29.41	131.15 ± 25.72	**0.002**
Tibial Tt.vBMD (mg HA/cm^3^)	293.67 ± 48.86	287.05 ± 58.5	0.474
Tibial Tb.vBMD (mg HA/cm^3^)	128.74 ± 37.65	142.97 ± 42.2	**0.037**
Tibial Ct.vBMD (mg HA/cm^3^)	944.01 ± 50.05	918.78 ± 63.04	**0.011**
Tibial Tb.BV/TV	0.206 ± 0.047	0.222 ± 0.055	0.063
Tibial Tb.N (1/mm)	1.108 (0.388)	1.166 (0.271)	**0.044^##^**
Tibial Tb.Th (mm)	0.268 ± 0.031	0.252 ± 0.024	**<0.001**
Tibial Tb.Sp (mm)	0.884 (0.32)	0.836 (0.211)	**0.011^##^**
Tibial Tb.1/N.SD (mm)	0.369 (0.159)	0.341 (0.107)	**0.019^##^**
Tibial Ct.Pm (mm)	105.15 ± 10.14	104.8 ± 12.14	0.857
Tibial Ct.Po (%)	0.016 ± 0.009	0.021 ± 0.012	**0.003^#^**
Tibial Ct.Th (mm)	1.617 ± 0.294	1.465 ± 0.281	**0.002**

Data are presented as mean ± SD or median (interquartile range).

Tt.Ar, total area; Tb.Ar, trabecular area; Ct.Ar, cortical area; Tt.vBMD, total volumetric bone mineral density; Tb.vBMD, trabecular volumetric bone mineral density; Ct.vBMD, cortical vBMD; Tb.N, trabecular number; Tb.Th, trabecular thickness; Tb.Sp, trabecular separation; Tb.1/N.SD, inhomogeneity of trabecular network; Tb.BV/TV, trabecular bone volume fraction (bone volume/tissue volume); Ct.Pm, cortical perimeter; Ct.Th, cortical thickness; Ct.Po, cortical porosity; HR-pQCT, high-resolution peripheral quantitative computed tomography.

^#^p-Value calculated after ln-transformation.

^##^p-Value calculated by Mann-Whitney U test.

Bold: p-Value < 0.05.

Referring to the distal tibia (**Table 2**), acromegaly patients presented a higher Ct.vBMD (944.01 ± 50.05 *vs.* 918.78 ± 63.04 mg HA/cm^3^, p *=* 0.011), Ct.Ar (145.57 ± 29.41 *vs.* 131.15 ± 25.72 mm^2^, p *=* 0.002), Ct.Th (1.617 ± 0.294 *vs.* 1.465 ± 0.281 mm, p *=* 0.002), Tb.Th (0.268 ± 0.031 *vs.* 0.252 ± 0.024 mm, p < 0.001), Tb.Sp [0.884 (0.32) *vs.* 0.836 (0.211) mm, p *=* 0.011], and Tb.1/N.SD [0.369 (0.159) *vs.* 0.341 (0.107) mm, p *=* 0.019]. Similarly, these patients had significantly decreased Tb.vBMD (128.74 ± 37.65 *vs.* 142.97 ± 42.2 mg HA/cm^3^, p *=* 0.037) and Tb.N [1.108 (0.388) *vs.* 1.166 (0.271)/mm, p *=* 0.044]. Surprisingly, acromegaly patients had smaller Ct.Po than controls (0.016 ± 0.009 *vs.* 0.021 ± 0.012, p *=* 0.003).

### Disease Activity Affects Bone Turnover Markers in Acromegaly Patients

Acromegaly patients were classified into two groups according to disease activity. As shown in **Table 3**, there were 27 active patients, including 14 males and 13 females. Their average age was 44.4 ± 10.8 years, average BMI was 26.3 ± 3.6 kg/m^2^, and average fasting IGF-1 and GH contents were 2.03 ± 0.64 ULN and 9.9 ± 15.9 ng/ml, respectively. In addition, 28 patients including nine males and 19 females had controlled disease. Their average age was 45.6 ± 12.2 years, and their BMI was around 24.6 ± 3.9 kg/m^2^. Their average random GH and IGF-1 were 1.0 ± 0.9 ng/ml and 0.78 ± 0.34 ULN, respectively. Differences in age (p *=* 0.707), BMI (p *=* 0.11), and gender (p *=* 0.227) were not statistically significant between active and controlled patients. The gonadal status (p *=* 0.676), diabetic status (p *=* 0.916), smoking status (p > 0.999), and 25(OH)D level (p *=* 0.461) were also comparable between these two groups. Nevertheless, raw IGF-1 (p < 0.001), IGF-1/ULN (IGF-1 divided by the age-specific ULN, p < 0.001), and GH (p < 0.001) were remarkably higher in the active group.

**Table 3:** Clinical characteristics and bone turnover indices of active and stable acromegaly patients.

		Acromegaly patients		p-Value
		Active (n = 27)	Controlled (n = 28)	
Age (years)		44.4 ± 10.8	45.6 ± 12.2	0.707
Gender (M/F)		14/13	9/19	0.227
BMI (kg/m^2^)		26.3 ± 3.6	24.6 ± 3.9	0.11
Duration of disease (years)		12.4 ± 8.0	12.4 ± 8.2	0.985
Diabetes mellitus (Y/N)		10/17	11/17	0.916
Eugonad (Y/N)		11/16	14/14	0.676
Smoking (%)		8.33 (2/24)	11.11 (3/27)	>0.999
Vitamin D supplementation (%)		3.70 (1/27)	10.71 (3/28)	0.611
GH (ng/ml)		9.9 ± 15.9	1.0 ± 0.9	**<0.001^#^**
IGF-1 (ng/ml)		548.9 ± 182.4	204.5 ± 83.2	**<0.001^#^**
IGF-1/ULN		2.03 ± 0.64	0.78 ± 0.34	**<0.001^#^**
DXA results (%)	T-score ≤ −2.5SD	25 (1/4)	0 (0/10)	0.286
	−2.5SD < T-score < −1SD	25 (1/4)	30 (3/10)	>0.999
	Z-score ≤ −2SD	15.4 (2/13)	6.7 (1/15)	0.583
BMD (g/cm^2^)	Femoral neck	0.979 ± 0.124	0.965 ± 0.148	0.754
	Total femur	1.016 ± 0.111	0.996 ± 0.158	0.673
	Lumbar spine	1.186 ± 0.185	1.131 ± 0.164	0.334
Vertebral fracture (%)		7.1 (1/14)	10.5 (2/19)	>0.999
FT3 (pg/ml)		2.88 ± 0.51	2.72 ± 0.43	0.223
FT4 (ng/dl)		1.24 ± 0.20	1.15 ± 0.24	0.119
TSH (μIU/ml)		1.37 ± 1.93	1.69 ± 1.06	0.453
ACTH (pg/ml)		28.1 ± 14.26	31.04 ± 17.24	0.503
Cortisol (μg/dl)		11.83 ± 4.104	13.57 ± 4.813	0.163
PRL (ng/ml)		12.09 ± 14.71	9.29 ± 7.73	0.388
FSH (IU/L)		14.38 ± 23.11	17.88 ± 23.65	0.588
LH (IU/L)		6.441 ± 7.825	8.665 ± 11.23	0.408
ALT (U/L)		16.41 ± 12.53	18.11 ± 9.135	0.574
Alb (g/L)		42.0 (6)	46.0 (3)	**<0.001** ^##^
Cr (μmol/L)		60.7 ± 15.57	62.21 ± 14.14	0.713
HbA1c (%)		6.0 (1.6)	5.9 (0.6)	0.276^#^
Ca (mmol/L)		2.33 ± 0.10	2.36 ± 0.08	0.272
P (mmol/L)		1.38 (0.16)	1.3 (0.36)	**0.04^##^**
ALP (U/L)		78.0 ± 23.5	76.8 ± 21.6	0.85
PTH (pg/ml)		43.4 ± 18.0	46.9 ± 16.6	0.483
25(OH)D (ng/ml)		20.7 ± 8.8	19.0 ± 8.3	0.461
β-CTX (ng/ml)		0.768 ± 0.302	0.532 ± 0.293	**0.012**
PINP (ng/ml)		83.2 ± 38.4	47.1 ± 30.7	**0.002^#^**

Data are presented as mean ± SD or median (interquartile range).

GH, growth hormone; IGF-1, insulin-like growth factor-1; IGF-1/ULN, IGF-1/upper limit of normal; FT3, free triiodothyronine; FT4, free thyroxine; TSH, thyroid-stimulating hormone; ACTH, adrenocorticotropic hormone; PRL, prolactin; FSH, follicle-stimulating hormone; LH, luteinizing hormone; ALT, alanine transaminase; Alb, albumin; Cr, creatinine; HbA1c, glycosylated hemoglobin; Ca, serum calcium; P, phosphate; ALP, alkaline phosphatase; PTH, parathyroid hormone; 25(OH)D, 25-hydroxyvitamin D; β-CTX, β-C-terminal telopeptide of type 1 collagen; PINP, total procollagen I N-terminal peptide.

^#^p-Value calculated after ln-transformation.

^##^p-Value calculated by Mann–Whitney U test.

Bold: p-Value < 0.05.

With respect to the controlled patients, the active patients had significantly elevated β-CTX (0.768 ± 0.302 *vs.* 0.532 ± 0.293 ng/ml, p *=* 0.012), PINP (83.2 ± 38.4 *vs.* 47.1 ± 30.7 ng/ml, p *=* 0.002), and phosphorus [1.38 (0.16) *vs.* 1.3 (0.36) mmol/L, p *=* 0.04]. Nevertheless, serum Ca (2.33 ± 0.10 *vs.* 2.36 ± 0.08 mmol/L, p *=* 0.272), ALP (78.0 ± 23.5 *vs.* 76.8 ± 21.6 U/L, p *=* 0.85), 25(OH)D (20.7 ± 8.8 *vs.* 19.0 ± 8.3 ng/ml, p *=* 0.461), and PTH (43.4 ± 18.0 *vs.* 46.9 ± 16.6 pg/ml, p *=* 0.483) concentrations were not statistically significant.

The axial aBMD and peripheral vBMD, together with the bone microarchitecture variables, do not significantly differ between these two disease activity groups (**Table 4**), in either the distal radius or distal tibia.

**Table 4:** HR-pQCT parameters of the distal radius and tibial in controlled and active acromegaly patients.

Parameters	Active (n = 27)	Controlled (n = 28)	p-Value
Radial Tt.Ar (mm^2^)	294.56 ± 82.66	269.81 ± 67.92	0.238
Radial Tb.Ar (mm^2^)	206.76 ± 71.2	181.48 ± 62.76	0.176
Radial Ct.Ar (mm^2^)	91.77 ± 21.07	92.05 ± 22.64	0.963
Radial Tt.vBMD (mg HA/cm^3^)	371.01 ± 74.67	408.63 ± 97.92	0.123
Radial Tb.vBMD (mg HA/cm^3^)	102.57 ± 49.45	105.27 ± 40.37	0.828
Radial Ct.vBMD (mg HA/cm^3^)	939.22 ± 53.54	967.64 ± 60.94	0.077
Radial Tb.BV/TV	0.163 ± 0.061	0.165 ± 0.053	0.907
Radial Tb.N (1/mm)	0.979 ± 0.394	0.996 ± 0.303	0.865
Radial Tb.Th (mm)	0.237 ± 0.034	0.238 ± 0.02	0.967
Radial Tb.Sp (mm)	1.318 ± 0.836	1.147 ± 0.613	0.397
Radial Tb.1/N.SD (mm)	0.76 ± 0.705	0.625 ± 0.537	0.436
Radial Ct.Pm (mm)	74.86 ± 11.81	71.05 ± 9.652	0.204
Radial Ct.Po (%)	0.006 ± 0.005	0.006 ± 0.006	0.781
Radial Ct.Th (mm)	1.511 ± 0.281	1.613 ± 0.393	0.286
Radial Ct.Po.Dm (mm)	0.185 ± 0.045	0.178 ± 0.035	0.538
Radial bone stiffness (N/mm)	87,530.34 ± 20,951.69	86,640.78 ± 22,239.62	0.882
Radial bone failure load (N)	4,793.27 ± 1,117.7	4,737.8 ± 1,226.16	0.864
Tibial Tt.Ar (mm^2^)	729.28 ± 121.52	710.61 ± 139.72	0.606
Tibial Tb.Ar (mm^2^)	588.48 ± 108.91	571.22 ± 130.39	0.604
Tibial Ct.Ar (mm^2^)	146.34 ± 29.4	144.82 ± 29.4	0.851
Tibial Tt.vBMD (mg HA/cm^3^)	289.03 ± 45.88	298.14 ± 51.17	0.499
Tibial Tb.vBMD (mg HA/cm^3^)	129.41 ± 38.91	128.09 ± 36.38	0.899
Tibial Ct.vBMD (mg HA/cm^3^)	933.19 ± 47.07	954.43 ± 50.62	0.12
Tibial Tb.BV/TV	0.206 ± 0.048	0.205 ± 0.045	0.894
Tibial Tb.N (1/mm)	1.109 ± 0.266	1.089 ± 0.275	0.786
Tibial Tb.Th (mm)	0.271 ± 0.037	0.265 ± 0.024	0.505
Tibial Tb.Sp (mm)	0.97 ± 0.291	1.007 ± 0.395	0.698
Tibial Tb.1/N.SD (mm)	0.453 ± 0.24	0.52 ± 0.421	0.485
Tibial Ct.Pm (mm)	106.03 ± 9.503	104.3 ± 10.65	0.536
Tibial Ct.Po (%)	0.018 ± 0.01	0.015 ± 0.007	0.225
Tibial Ct.Th (mm)	1.613 ± 0.294	1.621 ± 0.294	0.915
Tibial Ct.Po.Dm (mm)	0.243 ± 0.045	0.229 ± 0.031	0.253^#^
Tibial bone stiffness (N/mm)	198,168.67 ± 47,369.84	196,921.04 ± 46,421.14	0.923
Tibial bone failure load (N)	10,796.81 ± 2,432.7	10,755.38 ± 2,449.25	0.951

Data are presented as mean ± SD or median (interquartile range).

Tt.Ar, total area; Tb.Ar, trabecular area; Ct.Ar, cortical area; Tt.vBMD, total volumetric bone mineral density; Tb.vBMD, trabecular vBMD; Ct.vBMD, cortical vBMD; Tb.N, trabecular number; Tb.Th, trabecular thickness; Tb.Sp, trabecular separation; Tb.1/N.SD, inhomogeneity of trabecular network; Tb.BV/TV, trabecular bone volume fraction; Ct.Pm, cortical perimeter; Ct.Th, cortical thickness; Ct.Po, cortical porosity; Ct.Po.Dm, cortical pore diameter; HR-pQCT, high-resolution peripheral quantitative computed tomography.

^#^p-Value calculated after ln-transformation.

### Associations Between Insulin-Like Growth Factor-1/Upper Limit of Normal, Raw Insulin-Like Growth Factor-1, Growth Hormone, and Bone Microstructure Parameters

As shown in **Supplementary Figures 1 and 2**, we observed potential correlation between IGF-1/ULN and FT4 (β = 0.305, p *=* 0.023), and also between patient age and disease duration (β = 0.408, p *=* 0.002). As displayed in **Table 5**, after adjustment for gender, gonadal status, disease duration, and FT4 in multiple linear regression, higher IGF-1/ULN was associated with lower bone stiffness (β = −0.238, p *=* 0.017) and bone failure load (β = −0.248, p *=* 0.012) in the distal tibia. In addition, radial bone microstructure parameters were not significantly correlated with IGF-1/ULN. Similar trends were observed between HR-pQCT variables and raw IGF-1 level (**Supplementary Table 1**). According to **Table 6**, after gender, gonadal status, age, disease duration, and their interaction term were controlled, elevated GH was associated with larger Ct.Po (β = 0.275, p *=* 0.033), Ct.Po.Dm (β = 0.281, p *=* 0.026), and Tb.Th (β = 0.493, p < 0.001) in the distal radius. As for the tibia, lower Tb.N (β = −0.249, 0.044) and Tb.Ar (β = −0.225. p *=* 0.031) were observed for patients with high GH. In addition, tibial Ct.Th (β = 0.344, p *=* 0.011) and Tb.Th (β = 0.395, p < 0.001) increased as GH went up. After further adjustment for serum Ca (a possible confounder), a negative association between GH and Tb.BV/TV [β = −0.267, 95% CI: (−0.523, −0.012), p *=* 0.041] emerged.

**Table 5:** Association between bone microstructure parameters and IGF-1/ULN after adjusting for gender, gonadal status, disease duration, and FT4, as well as the interaction between IGF-1/ULN and FT4.

Parameter	Standardized β	95% CI	p-Value (FT4 adjusted)	p-Value (BMI adjusted)
Tibial bone stiffness (N/mm)	−0.238	(−0.400, −0.042)	**0.017**	**0.001**
Tibial bone failure load (N)	−0.248	(−0.409, −0.054)	**0.012**	**<0.001**

p-Value (BMI adjusted): adjusted for gender, gonadal status, disease duration, and BMI, as well as the interaction between IGF-1/ULN and BMI.

Bold: p-Value < 0.05.

**Table 6:** Association between bone microstructure parameters and GH after adjusting for gender, gonadal status, disease duration, and age, as well as the interaction term between disease duration and age.

Parameter	Standardized β	95% CI	p-Value
Radial Tb.Th (mm)	0.493	(0.298, 0.688)	**<0.001**
Radial Ct.Po (%)	0.275	(0.023, 0.527)	**0.033**
Radial Ct.Po.Dm (mm)	0.281	(0.036, 0.527)	**0.026**
Tibial Tb.Ar (mm^2^)	−0.225	(−0.428, −0.021)	**0.031**
Tibial Tb.N (1/mm)	−0.249	(−0.490, −0.007)	**0.044**
Tibial Tb.Th (mm)	0.395	(0.200, 0.590)	**<0.001**
Tibial Ct.Th (mm)	0.344	(0.084, 0.605)	**0.011**

Tb.Th, trabecular thickness; Ct.Po, cortical porosity; Ct.Po.Dm, cortical pore diameter; Tb.Ar, trabecular area; Tb.N, trabecular number; Ct.Th, cortical thickness; GH, growth hormone.

Bold: p-Value < 0.05.

We further analyzed bone microarchitecture parameters in each gender after controlling for disease duration and gonadal status (replaced by raw testosterone level in the male group). For males (**Table 7**), IGF-1/ULN was negatively associated with radial Tb.N (β = −0.392, p *=* 0.031) and positively correlated with Tb.Sp (β = 0.374, p *=* 0.014). In the radial cortical bone, Ct.Po (β = 0.489, p *=* 0.001) and Ct.Po.Dm (β = 0.579, p *=* 0.002) increased as IGF-1/ULN rose. In the distal tibia, Tt.Ar (β = −0.347, p = 0.028) and Tb.Ar (β = −0.412, p *=* 0.018) dropped as IGF-1/ULN surged. Additionally, IGF-1/ULN was a positive predictor for tibial Tb.Th (β = 0.386, p *=* 0.046). When IGF-1/ULN was replaced by IGF-1 in the regression models, the results were largely similar, except for the emergence of a negative relationship between raw IGF-1 and tibial Tb.N (β = −0.373, p *=* 0.047, shown in **Supplementary Table 2**). Furthermore, males with higher GH typically had larger Tb.Th (β = 0.624, p < 0.001) and bigger Ct.Po (β = 0.406, p < 0.001) and Ct.Po.Dm (β = 0.321, p *=* 0.049) in the radius. Moreover, the increase in GH was correlated with thicker tibia with larger Tb.Th (β = 0.513, p < 0.001) and Ct.Th (β = 0.431, p *=* 0.002). Tibial Ct.Ar (β = 0.271, p *=* 0.036) also rose as GH increased (**Supplementary Table 3**). Conversely, in the female group, all the HR-pQCT parameters were not significantly altered as IGF-1/ULN, IGF-1, or GH increased.

**Table 7:** Association between bone microstructure parameters and IGF-1/ULN in males after adjusting for disease duration and testosterone level.

Parameter	Standardized β	95% CI	p-Value
Radial Tb.N (1/mm)	−0.392	(−0.744, −0.040)	**0.031**
Radial Tb.Sp (mm)	0.374	(0.083, 0.664)	**0.014**
Radial Ct.Po (%)	0.489	(0.234, 0.744)	**0.001**
Radial Ct.Po.Dm (mm)	0.579	(0.246, 0.912)	**0.002**
Tibial Tt.Ar (mm^2^)	−0.347	(−0.652, −0.041)	**0.028**
Tibial Tb.Ar (mm^2^)	−0.412	(−0.746, −0.079)	**0.018**
Tibial Tb.Th (mm)	0.386	(0.008, 0.763)	**0.046**

Tb.N, trabecular number; Tb.Sp, trabecular separation; Ct.Po, cortical porosity; Ct.Po.Dm, cortical pore diameter; Tt.Ar, total area; Tb.Ar, trabecular area; Tb.Th, trabecular thickness; IGF-1, insulin-like growth factor-1; ULN, upper limit of normal.

Bold: p-Value < 0.05.

## Discussion

Acromegaly patients suffer from a higher incidence of vertebral fractures and accelerated bone turnover due to excessive GH and IGF-1 production (4). Specifically, DXA was commonly used to measure the aBMD in the femoral neck, lumbar spines, and total hip, thereby facilitating the diagnosis of osteoporosis and fracture risk prediction. In addition, active acromegaly patients exhibited an up to eight-fold increased rate of fragility fractures due to compromised bone quality rather than bone quantity (20). Moreover, vertebral fractures still happened in 20% well-controlled acromegaly patients with no osteopenia or osteoporosis (3). Using impact micro-indentation, Malgo et al. illustrated that acromegaly patients in remission have impaired cortical bone strength relative to healthy controls (21). Furthermore, the trabecular bone score (TBS) of acromegaly patients continues to decline after the disease had been rigorously controlled (22, 23). Therefore, we should pay more attention to evaluating bone microstructure and preventing fractures in acromegaly patients, even if the disease had been satisfactorily controlled. Stratifying patients into different groups according to their fracture risks is important, so proactive anti-osteoporotic therapies could be initiated in a timely and individualized manner.

Hitherto, the vertebral fracture incidence among the Chinese acromegaly cases had rarely been reported. In this study, we used DXA to assess axial aBMD and thoracolumbar spine X-ray to spot vertebral fractures in acromegaly patients. Among 14 patients who were more than 50-year-old (male)/menopausal (female), one case (7.1%) was diagnosed as osteoporosis and four cases (28.6%) as osteopenia. In addition, in males younger than 50 years and females before menopause, there were three patients (10.7%) whose Z-score was ≤−2 SD. As for the 33 patients who underwent thoracolumbar spine X-ray examination, three cases (9.1%) had vertebral fractures without relevant symptoms. Two of them had normal BMD, and one case was a postmenopausal woman with osteopenia (T-score in lumbar spine: −2.2). The vertebral fracture rate in the present work was lower than that reported by previous studies (around 40%) due to the limited sample size (24).

As bone quality is highly dependent on 3D bone structure, aBMD measurements obtained through DXA failed to accurately reflect the bone fragility of acromegaly patients. In particular, DXA is unable to differentiate cortical bone from trabecular bone. However, the axial bone strength mainly relies on trabecular BMD rather than cortical BMD. As acromegaly patients have decreased trabecular BMD but increased cortical BMD, they may have approximately normal DXA measurements even when trabecular bone had been considerably impaired (25). The shortcomings of DXA may be overcome by HR-pQCT, in which bone microarchitecture variables and vBMD are assessed (26). To date, the present work is the first to evaluate the bone microstructure of Chinese acromegaly patients through HR-pQCT. We detected obvious changes in cortical (elevated Ct.Ar, Ct.Th, and Ct.vBMD) as well as trabecular (elevated Tb.Sp and network inhomogeneity; reduced Tb.vBMD, Tb.BV/TV, and Tb.N) microarchitecture of the distal tibia and radius among acromegaly cases relative to normal controls. Nevertheless, the Ct.Ar, Ct.Th, and Ct.vBMD had increased, which could be attributed to intensified periosteal ossification induced by excessive GH exposure (27).

To further evaluate how disease activity affected bone turnover markers and microstructure, the acromegaly patients were divided into two groups based on their disease activity. These two groups were comparable at baseline in terms of age, BMI, disease duration, gender ratio, gonadal status, and diabetic status. We found that the active group had significantly increased β-CTX, PINP, and serum phosphorus, which indicate accelerated bone turnover. Nevertheless, serum calcium, 25(OH)D, ALP, and PTH did not differ by much in these two groups. With regard to the axial aBMD, peripheral vBMD, and bone microstructure parameters, no significant differences were found between the active and controlled groups. Namely, although active acromegaly patients had accelerated bone reabsorption, their bone quality and strength in the extremities were not remarkably inferior to those of controlled patients. One possible explanation is that HR-pQCT was performed soon after biochemical alleviation was achieved in some patients. Specifically, the time in remission spans from 1 month to 9 years 10 months in the controlled group. Although the speed of bone reabsorption had already slowed down, some patients had little time for bone structure restoration before being enrolled in this study. It is also possible that the damages resulted from elevated GH/IGF-1 might not be fully reversible, especially for patients with longer active disease duration before finally achieving remission. In particular, controlled acromegaly patients were likely to suffer from severe osteoblast dysfunction, as both osteoblast vigor and osteoblast number were significantly compromised in controlled patients (28).

We further investigated the relation between bone fragility and the hyperactivity of the GH/IGF-1 axis through multiple linear regression after examining the collinearity of potential independent variables. After gender, disease duration, FT4, and gonadal status were controlled, acromegaly patients with higher IGF-1/ULN had lower tibial failure load and stiffness relative to controlled patients. As tibia is important for weight-bearing, the reduction in its strength may result in dangerous falls ensued by bone fractures. Moreover, GH was a positive predictor for radial Ct.Po, as well as a negative predictor for tibial Tb.N and bone volume fraction. Subgroup analysis revealed that male patients with high IGF-1/ULN suffered from declined Tb.N, together with increased Tb.Sp and cortical pore diameter in the distal radius. Moreover, we observed decreased Tt.Ar and Tb.Ar in the tibia among males.

These results indicated that patients exposed to higher levels of IGF-1/GH may have more pronounced deterioration in bone strength and quality, underpinning the importance of maintaining biochemical remission in acromegaly treatment. Nevertheless, patients with higher GH had thicker cortical and trabecular bone, which may partially compensate for the impaired bone structure. However, the activity of the GH/IGF-1 axis was not significantly associated with HR-pQCT parameters that are most essential for bone quality, such as Tb.vBMD (in regression model for IGF-1/ULN, radial: β = −0.105, p *=* 0.457; tibial: β = −0.206, p *=* 0.111). Hence, well-controlled acromegaly patients may still have poor bone quality. Anti-osteoporotic therapy may be necessary even after biochemical alleviation had been achieved to decrease bone fracture risk and improve life quality of acromegaly patients. The anti-osteoporotic therapy for an acromegaly patient may be tailored by his/her risk for fragility fractures.

Certain limitations should be noted for the present work. First of all, clinical data for the healthy controls other than age, gender, and BMI were not available. Hence, factors that may affect bone strength, including the smoking status, gonadal status, and serum 25(OH)D level, might not be comparable between acromegaly patients and healthy controls. Secondly, this study had a limited sample of 55 acromegaly patients and 110 healthy controls. Due to the small sample size, this study might not reveal all bone microstructure changes that could occur in acromegaly patients. Another pitfall lies in its nature as an exploratory cross-sectional study. Prospective cohort studies that measure the bone microstructure parameters of acromegaly patients before and after treatment will provide more convincing evidence for the changes in bone microstructure following biochemical remission. Additionally, simply regarding the gonadal status as a bicategorical variable may obscure the differences between naturally eugonad people and patients on sex hormone replacement therapy. Moreover, this study did not account for factors such as diet composition, exercise, and other factors that could affect the bone health of patients. Furthermore, as only 33 patients were assessed by the thoracolumbar spine X-ray, we did not have enough samples to explore the relationship between the incidence of vertebral fractures and acromegaly disease activity. An integrated model taking both bone turnover markers and microarchitecture parameters into consideration may better facilitate bone fracture risk prediction and guide informed clinical decision making for anti-osteoporotic therapy.

## Conclusion

The following conclusions can be drawn for this study: 1) as compared with healthy controls, acromegaly patients had remarkably deteriorated bone microstructure, especially for the trabecular bone; 2) although suppressing the hyperactive GH/IGF-1 axis in acromegaly patients decreased bone reabsorption, the improvements on bone quality were quite limited. Although patients with elevated GH/IGF-1 had higher radial cortical bone porosity, fewer tibial trabeculae, and lower tibial failure load, their bone quality was partially compensated by increased bone thickness. 3) Controlling GH/IGF-1 might help protect the bone strength in males. 4) HR-pQCT has the potential to serve as a clinical tool for assessing bone microarchitecture in acromegaly patients. Therefore, clinicians should be aware of the persistent bone quality deterioration in controlled acromegaly patients and be prepared for initiating anti-osteoporotic therapy in those patients.

## Data Availability Statement

The raw data supporting the conclusions of this article will be made available by the authors, without undue reservation.

## Ethics Statement

The studies involving human participants were reviewed and approved by Institutional Review Committee of Peking Union Medical College Hospital. The patients/participants provided their written informed consent to participate in this study.

## Author Contributions

Study conception and design: HZ, WX, LD, and SY. Participants’ recruitment and data collection: LD, SY, RL, YXZ, and HD. Image quality control and analysis: LD, HY, XZ, and RL. Statistical analysis: LD, YLZ, and SY. Manuscript drafting: LD and SY. Manuscript revision: HZ, WX, and FG. All authors contributed to the article and approved the submitted version.

## Funding

The present study was funded by the National Key Research and Development Program of China (No. 2016YFC091501) and the CAMS Innovation Fund for Medical Science (CAMS-2016-I2M-1-002).

## Conflict of Interest

The authors declare that the research was conducted in the absence of any commercial or financial relationships that could be construed as a potential conflict of interest.

## Publisher’s Note

All claims expressed in this article are solely those of the authors and do not necessarily represent those of their affiliated organizations, or those of the publisher, the editors and the reviewers. Any product that may be evaluated in this article, or claim that may be made by its manufacturer, is not guaranteed or endorsed by the publisher.
