# Supplementary material for: The Negative Impacts of Acromegaly on Bone Microstructure Not Fully Reversible
Source: Front Endocrinol (Lausanne). 2021 Sep 15;12:738895. doi: 10.3389/fendo.2021.738895 (PMC8479105; doi:10.3389/fendo.2021.738895)
Supplement: Supplementary file 1 [file DataSheet_1.docx]

Supplementary Material

# Supplementary Tables

**Supplementary Table 1**. Association between bone microstructure parameters and raw IGF-1 in all subjects after adjusting for gender, gonad status, age, disease duration, as well as interaction between disease duration and age.

| Parameter | Standardized β | 95% CI | P value | P value (FT4 adjusted) |
| --- | --- | --- | --- | --- |
| Tibial Bone stiffness (N/mm) | -0.261 | (-0.434, -0.088) | **0.004** | **0.007** |
| Tibial Bone failure load (N) | -0.268 | (-0.439, -0.097) | **0.003** | **0.004** |

P value (FT4 adjusted): P value obtained after adjusting for gender, gonad status, age, disease duration, interaction between disease duration and age, FT4, as well as interaction between IGF-1 and FT4.

**Supplementary Table 2**. Association between bone microstructure parameters and raw IGF-1 in males after adjusting for testosterone level, age, disease duration, as well as interaction between disease duration and age.

| Parameter | Standardized β | 95% CI | P value |
| --- | --- | --- | --- |
| Radial Tb.N (1/mm) | -0.375 | (-0.722, -0.027) | **0.036** |
| Radial Tb.Sp (mm) | 0.362 | (0.052, 0.671) | **0.025** |
| Radial Ct.Po (%) | 0.469 | (0.202, 0.736) | **0.002** |
| Radial Ct.Po.Dm (mm) | 0.555 | (0.182, 0.928) | **0.006** |
| Tibial Tb.Ar (mm^2^) | -0.426 | (-0.780, -0.071) | **0.022** |
| Tibial Tb.N (1/mm) | -0.373 | (-0.741, -0.005) | **0.047** |

Trabecular number (Tb.N), trabecular separation (Tb.Sp), cortical porosity (Ct.Po), cortical pore diameter (Ct.Po.Dm), trabecular area (Tb.Ar).

**Suplementary Table 3**. Association between bone microstructure parameters and GH in males after adjusting for testosterone level, age, disease duration, as well as interaction between disease duration and age.

| Parameters | Standardized β | 95% CI | P value |
| --- | --- | --- | --- |
| Radial Tb.Th (mm) | 0.624 | (0.424, 0.825) | **<0.001** |
| Radial Ct.Po (%) | 0.406 | (0.225, 0.587) | **<0.001** |
| Radial Ct.Po.Dm (mm) | 0.321 | (0.001, 0.641) | **0.049** |
| Tibial Ct.Ar (mm) | 0.271 | (0.019, 0.522) | **0.036** |
| Tibial Tb.Th (mm) | 0.513 | (0.276, 0.749) | **<0.001** |
| Tibial Ct.Th (mm) | 0.431 | (0.180, 0.682) | **0.002** |

Trabecular thickness (Tb.Th), cortical porosity (Ct.Po), cortical pore diameter (Ct.Po.Dm), cortical area (Ct.Ar), cortical thickness (Ct.Th).

# Supplementary Figures

**Supplementary Figure 1.** Pearson Correlation coefficients of potential independent variables after ln-transformation.

BMI, bone mass index; GH, growth hormone; IGF-1, insulin-like growth factor-1; IGF-1/ULN, IGF-1/upper limit of normal; FSH: follicle stimulating hormone; LH, luteinizing hormone; PRL, prolactin; ACTH, adrenocorticotropic hormone; FT3, free triiodothyronine; FT4, free thyroxine; TSH, thyroid stimulating hormone; ALT, alanine transaminase; Cr, creatinine, Alb, albumin; Glu, blood glucose; Ca, blood calcium; 25(OH)D, 25 hydroxyvitamin D.


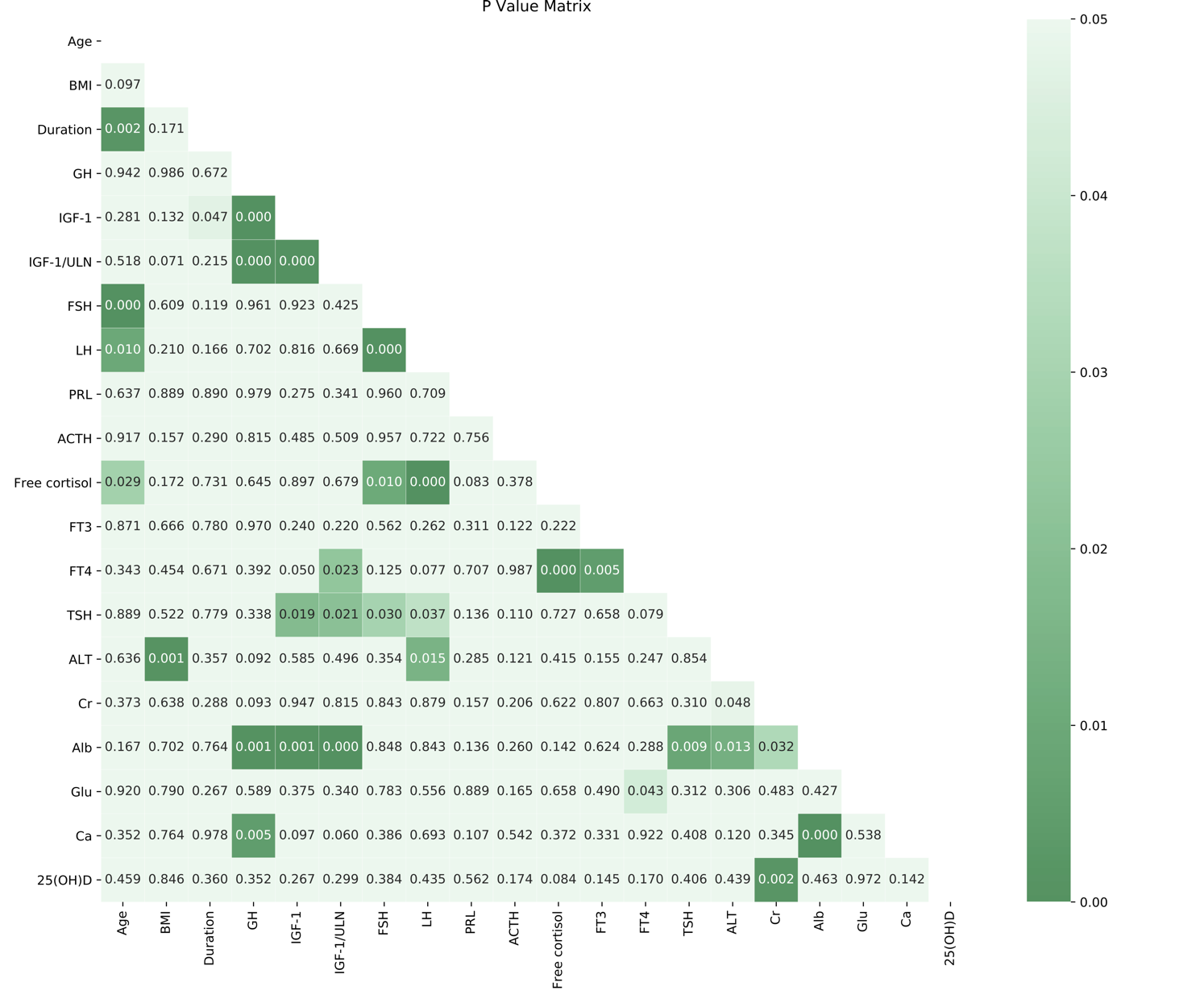


**Supplementary Figure 2.** P values for Pearson Correlation coefficients of potential independent variables after ln-transformation.

BMI, bone mass index; GH, growth hormone; IGF-1, insulin-like growth factor-1; IGF-1/ULN, IGF-1/upper limit of normal; FSH: follicle stimulating hormone; LH, luteinizing hormone; PRL, prolactin; ACTH, adrenocorticotropic hormone; FT3, free triiodothyronine; FT4, free thyroxine; TSH, thyroid stimulating hormone; ALT, alanine transaminase; Cr, creatinine, Alb, albumin; Glu, blood glucose; Ca, blood calcium; 25(OH)D, 25 hydroxyvitamin D.
